# Use of the MOF NU-1000 as a Drug Delivery System for the Antineoplastic Drug Mitoxantrone

**DOI:** 10.3390/ijms27114857

**Published:** 2026-05-28

**Authors:** Daniel R. Alfonso, Francisco G. Moscoso, David Rodríguez-Lucena, Javier Roales, Carolina Carrillo-Carrión, María Victoria Cascajo-Almenara, Carlos Santos-Ocaña, José M. Pedrosa

**Affiliations:** 1 Center for Nanoscience and Sustainable Technologies (CNATS), Department of Physical, Chemical and Natural Systems, Universidad Pablo de Olavide, 41013 Sevilla, Spain; druialf@alu.upo.es (D.R.A.); fjgarmos@upo.es (F.G.M.); javier.roales@gmail.com (J.R.); 2 Departamento de Química Orgánica y Farmacéutica, Facultad de Farmacia, Universidad de Sevilla, 41012 Sevilla, Spain; 3 Institute for Chemical Research (IIQ), CSIC-University of Seville, 41092 Sevilla, Spain; carolina.carrillo@csic.es; 4 Andalusian Center for Developmental Biology, University Pablo de Olavide, CIBERER, 41013 Sevilla, Spain; mvcasalm@upo.es (M.V.C.-A.); csanoca@upo.es (C.S.-O.)

**Keywords:** mitoxantrone, NU-1000, metal–organic frameworks, drug delivery, PEGylation, cytotoxicity

## Abstract

Metal–organic frameworks (MOFs) offer unique opportunities for drug delivery due to their high porosity and the possibility of hosting large drug molecules within well-defined pore systems. In this work, the zirconium-based MOF NU-1000 was investigated as a carrier for the antineoplastic drug mitoxantrone (MTX). NU-1000 particles were synthesized and characterized by PXRD, SEM, and DLS, confirming their crystallinity, morphology, and size distribution. MTX loading was achieved by aqueous incubation and quantified by UV-Vis spectroscopy and thermogravimetric analysis, yielding a high loading capacity of ~40–43 wt%, with most of the uptake occurring within the first three hours. Structural characterization demonstrated that the MOF preserves its crystallinity and morphology after drug incorporation, while the DLS results suggest that MTX is mainly accommodated within the internal pore system. To improve stability under physiological conditions, the composite was coated with NH_2_-PEG-NH_2_, resulting in PEG@MTX@NU-1000 particles with enhanced stability in phosphate-buffered saline. Cytotoxicity assays in HeLa cells showed that the PEGylated carrier is largely biocompatible, while PEG@MTX@NU-1000 exhibits a significantly enhanced antiproliferative effect compared to free MTX at short incubation times. These results demonstrate that NU-1000 is a promising platform for MTX delivery, combining high loading capacity, structural stability after PEGylation, and improved short-term therapeutic performance.

## 1. Introduction

Cancer remains one of the leading causes of mortality worldwide [1] and chemotherapy continues to be one of the main therapeutic strategies used to treat it through the administration of cytotoxic drugs that interfere with cell growth and division [2]. Although these agents are generally more effective against rapidly proliferating cancer cells, they also affect healthy tissues, leading to significant side effects [3]. This limitation has driven the development of alternative strategies aimed at reducing systemic toxicity while maintaining therapeutic efficacy. Among these, drug delivery systems based on encapsulation have emerged as a promising approach, enabling controlled drug release and improved therapeutic performance [4].

In this context, nanomedicine has provided a wide range of drug carriers based on biocompatible materials, designed to overcome some of the main drawbacks of conventional chemotherapy, such as short circulation time, low bioavailability, and limited cellular uptake [5,6,7]. These nanoscale systems can be engineered to improve drug transport and biodistribution, and in some cases to achieve targeted delivery. For instance, functionalization with targeting ligands such as peptides, proteins, or nucleic acids enables selective interactions with specific tissues [8,9], while surface modification with polyethylene glycol (PEG) has been widely employed to increase circulation time and reduce nonspecific interactions [10,11].

Despite these advances, the development of efficient drug delivery systems remains challenging, since not all existing platforms simultaneously fulfill key requirements such as biocompatibility, precise control over physicochemical properties governing cellular uptake, and scalable synthesis methods suitable for practical applications [7,12,13].

In this regard, metal–organic frameworks (MOFs) have emerged as a particularly attractive class of materials for drug delivery due to their high porosity, large surface area, and structural tunability, which enable the efficient encapsulation and transport of a wide variety of molecules [14,15,16]. Their development in biomedicine has progressed from an initial proof of concept to a well-established research field, particularly after early studies with biocompatible MOFs, especially iron-carboxylate systems, demonstrated that these materials could combine unusually high payloads with acceptable tolerance in biological media, thereby distinguishing them from conventional carriers with much lower loading capacities [17,18,19]. Since then, MOFs have been intensively investigated as drug delivery platforms because their pore size, connectivity, composition, and surface chemistry can be tailored to control guest uptake, host–guest interactions, and release behavior [14,15,19].

This structural versatility has been particularly valuable for the delivery of anticancer agents, for which high loading, protection of the cargo, and controllable release are critical requirements [14]. MOF-based systems have been shown to support the encapsulation of chemotherapeutics, the engineering of stimulus-responsive release profiles, and the integration of surface modifications that improve colloidal behavior and biological performance [14,20,21,22].

Among clinically relevant antineoplastic agents, mitoxantrone (MTX) is of particular interest. This synthetic anthracenedione is widely used in the treatment of breast and prostate cancers, leukemias, and lymphomas, and exerts its antitumor activity mainly through DNA intercalation and topoisomerase II poisoning [23]. However, its therapeutic application remains limited by nonspecific biodistribution and dose-dependent systemic toxicity, including clinically relevant cardiotoxicity, which makes encapsulation strategies especially attractive for improving its therapeutic index [23,24].

Consistent with this rationale, several MOF-based systems have already been investigated for mitoxantrone delivery. Reported examples include Fe/phytic-acid nanoMOFs for bone-metastatic prostate cancer immunotherapy, zirconium-based UiO-66 and UiO-66-NH_2_ for controlled MTX encapsulation and release, β-cyclodextrin-modified redox/pH-responsive MOFs for targeted breast-cancer therapy, and more complex biomimetic co-loading platforms that use mitoxantrone as part of combination regimens [25,26,27,28]. These studies collectively support the feasibility of incorporating mitoxantrone into porous frameworks, while also showing that loading capacity, colloidal behavior, and stability remain strongly dependent on the specific MOF platform.

Among zirconium-based candidates, NU-1000 is particularly attractive for drug delivery because it combines high chemical robustness with a hierarchically porous structure containing large mesoporous channels and accessible Zr_6_ nodes suitable for post-synthetic surface modification [29,30]. NU-1000 has already shown promise in biomedical cargo delivery, including acid-resistant insulin encapsulation and release in an oral delivery context, as well as chemotherapy-related transport of doxorubicin [31,32]. These precedents support its evaluation as a platform for mitoxantrone encapsulation and delivery.

In this work, we investigate the zirconium-based MOF NU-1000 as a carrier for the antineoplastic drug mitoxantrone, with the aim of assessing its capacity for drug incorporation, the preservation of the framework after loading, and the effect of PEGylation on the stability of the system under physiologically relevant conditions. In addition, the biological performance of the resulting formulation is examined in order to determine whether this platform can improve the antiproliferative activity of MTX compared to the free drug.

## 2. Results and Discussion

### 2.1. Synthesis and Characterization of NU-1000

**Figure 1:**
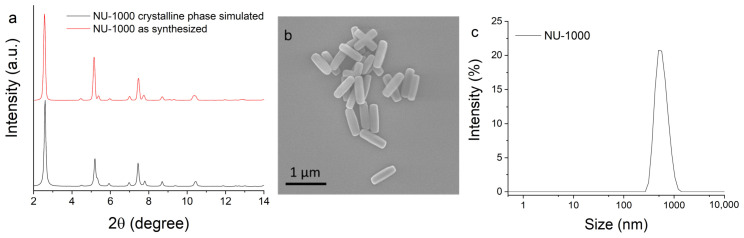
(**a**) PXRD pattern of the synthesized NU-1000 together with the simulated pattern (COD 7230579) for comparison. (**b**) SEM micrograph of the synthesized NU-1000 crystals. (**c**) DLS size distribution of NU-1000 particles dispersed in water.

The NU-1000 particles, isolated as a crystalline pale-yellow powder, were characterized by powder X-ray diffraction (PXRD), scanning electron microscopy (SEM), and dynamic light scattering (DLS), and the corresponding results are shown in Figure 1. The PXRD diffractogram of the synthesized material (Figure 1a) reveals high crystallinity, and its good agreement with the simulated pattern confirms the phase purity of the obtained NU-1000. SEM images (Figure 1b) show rod-shaped crystals with sizes of approximately 600–700 nm, with some particles exhibiting twinning that results in characteristic cross-shaped morphologies. Overall, the images indicate the absence of significant aggregation and a relatively homogeneous particle size distribution, which is further supported by DLS measurements after dispersion in water. As shown in Figure 1c, the DLS data display a narrow size distribution with an average hydrodynamic diameter of 663 nm, in good agreement with the particle sizes observed by SEM, and a polydispersity index (PDI) of 0.095, indicating a reasonably uniform particle population.

### 2.2. Mitoxantrone Loading into NU-1000

MTX in phosphate-buffered saline (PBS 1×, pH 7.4) was quantified by UV-Vis spectrophotometry. The corresponding absorption spectra at different concentrations are shown in Figure S1a. Three features are observed at 661, 609, and 565 nm, consisting of two main peaks and a shoulder. These bands arise from a single electronic transition that appears split due to π-π stacking interactions between the anthraquinone rings, and lies in the visible region as a result of a charge-transfer transition from the amino substituent to the anthraquinone ring itself [33]. Specifically, the band at 661 nm is assigned to the monomer, the band at 609 nm to the dimer, and the shoulder at 565 nm to higher-order aggregates [34]. These aggregates adopt geometries that can be described within the molecular exciton model. Consistently, the intensity of the aggregate bands increases with concentration, while the monomer band decreases. This concentration-dependent change in the MTX spectral profile (i.e., the extinction coefficient at a given wavelength) makes the direct application of the Beer-Lambert law at a single wavelength unsuitable for quantification. However, the oscillator strength of the transition is proportional to the integrated area of the absorption band ε(λ), which remains constant regardless of the monomer-dimer equilibrium [35]. Therefore, the integrated area of the MTX absorption bands in the 500–750 nm range maintains a linear relationship with concentration and can be used to construct a calibration curve. The resulting calibration plot (Figure S1b) shows excellent linearity and was used to determine the MTX concentration in solution during the loading experiments.

Drug loading was carried out by incubating NU-1000 particles with an aqueous MTX solution under the conditions described in the Experimental section. After incubation, the initially pale-yellow NU-1000 particles turned deep blue and retained this coloration even after extensive washing with water, indicating a strong retention of MTX within the material. The extent of drug incorporation was quantified by UV-Vis spectroscopy by monitoring the decrease in MTX absorbance in the supernatant. Figure S2a shows the progressive decrease in the characteristic MTX absorption bands in the solution, which allows the amount of drug incorporated into the MOF to be determined. The loading capacity (LC, %), expressed as mg of MTX per mg of NU-1000 ×100, reached values of approximately 43%, indicating a high drug uptake by the material. From a kinetic perspective, the results shown in Figure S2b reveal that most of the MTX incorporation occurs within the first three hours, suggesting a relatively fast inclusion process. Consistently, the percentage of MTX uptake from solution increased from 40.4% at 10 min to 69.4% at 3 h, reaching 86.0% after 24 h (Table S1).

**Figure 2:**
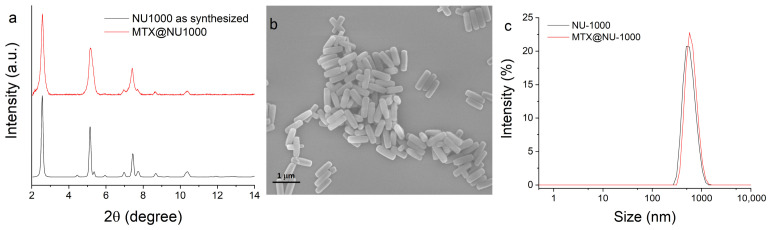
(**a**) PXRD pattern of MTX@NU-1000 compared with that of pristine NU-1000. (**b**) SEM micrograph of MTX@NU-1000 crystals. (**c**) DLS size distribution of MTX@NU-1000 particles dispersed in water, compared to pristine NU-1000.

The MTX@NU-1000 composite was characterized to evaluate whether the MOF structure and morphology were preserved after drug incorporation (Figure 2). The PXRD pattern of MTX@NU-1000 (Figure 2a) shows an excellent agreement with that of pristine NU-1000, indicating that the crystalline structure and topology are maintained after MTX loading. Likewise, SEM images (Figure 2b) reveal that the particles retain their characteristic rod-like morphology, with no observable changes in shape, confirming that the inclusion process does not affect the external structure of the MOF crystals. Although some local particle clustering may be observed in the SEM micrograph, this is attributed to solvent evaporation during sample preparation for SEM, whereas the DLS results indicate that MTX@NU-1000 remains reasonably well dispersed in aqueous suspension.

DLS measurements (Figure 2c) show an average hydrodynamic diameter of 708 nm, slightly higher than that of pristine NU-1000 (663 nm), while maintaining a similar polydispersity index (PDI ≈ 0.107 vs. 0.095). This modest increase in size, together with the preservation of morphology and crystallinity, suggests that MTX incorporation occurs mainly within the internal pore system of NU-1000 rather than through significant adsorption on the external surface.

The preferential incorporation of MTX within the internal pore system of NU-1000 is likely favored by specific host–guest interactions between the drug and the framework. In particular, π-π stacking interactions between the anthraquinone core of MTX and the extended aromatic TBAPy linker of NU-1000 are expected to play a dominant role in stabilizing the guest molecules within the pores, as previously reported for other aromatic compounds [36,37]. Additionally, the relatively hydrophobic character of both MTX and the pyrene-based linkers may promote encapsulation through solvophobic effects, further enhancing host–guest affinity. These synergistic non-covalent interactions can explain the high loading capacity observed and the strong retention of MTX within the MOF, in agreement with previous studies reporting strong guest confinement in NU-1000 [38,39].

The presence of MTX in the MTX@NU-1000 composite was further confirmed by UV-Vis spectroscopy (Figure S3). The absorption spectrum of the MTX@NU-1000 suspension exhibits the characteristic bands of MTX, in good agreement with those observed for the free drug in aqueous solution. In addition, the spectral features associated with the NU-1000 framework (TBAPy linker) are also present in the composite spectrum.

**Figure 3:**
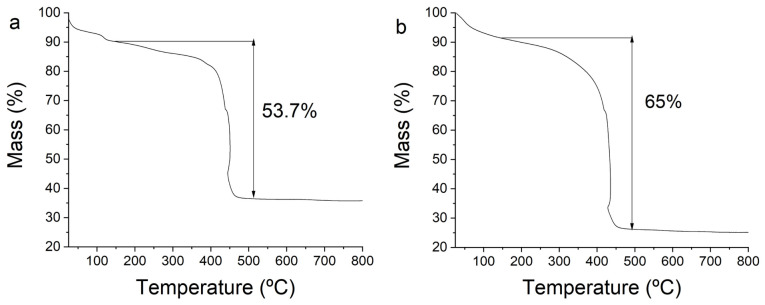
(**a**) TGA curve of pristine NU-1000 and (**b**) MTX@NU-1000, showing the different mass loss profiles used for loading capacity determination.

Thermogravimetric analysis (TGA) was employed as a direct method to quantify the MTX loading in NU-1000 (Figure 3). The TGA profiles of pristine NU-1000 and MTX@NU-1000 (Figure 3a,b) show similar decomposition patterns. An initial weight loss below ~400 °C is observed in both samples, which can be attributed to the removal of adsorbed species (water and residual solvents). At higher temperatures, a sharp mass loss is detected, corresponding to the decomposition of the organic linker, followed by the formation of a residual inorganic phase assigned to ZrO_2_.

Despite the similarity in the decomposition profiles, a clear difference in the total weight loss is observed between both samples. Pristine NU-1000 exhibits a total mass loss of 53.7%, whereas MTX@NU-1000 shows a significantly higher value of 65%, which can be attributed to the presence of the encapsulated MTX.

To quantify the drug loading, the TGA curve of pristine NU-1000 was first used to determine the experimental metal-to-linker molar ratio. A value of n(Zr)/n(TBAPy) = 3.7 was obtained, slightly higher than the theoretical value (3), which can be attributed to the presence of structural defects associated with linker deficiency. Assuming that the Zr/TBAPy ratio remains constant after MTX loading, the additional mass loss observed in MTX@NU-1000 was attributed to the presence of MTX, which allows the amount of incorporated drug to be quantified. A detailed description of the calculation is provided in the Supplementary Material. Based on this analysis, the loading capacity determined by TGA was estimated to be 39 wt%, in very good agreement with the value obtained by UV-Vis spectroscopy.

The loading capacity obtained for MTX in NU-1000 (~40–43 wt%) is comparable to the highest values reported for zirconium-based MOFs such as UiO-66 [26], and exceeds those reported for several other MOF-based systems [25,28]. Although higher loading capacities have been described for β-cyclodextrin-based MOFs (~66%) [27], these materials typically exhibit limited structural stability in aqueous environments. In this context, NU-1000 offers relevant advantages over more conventional Zr-based UiO-type carriers. Its hierarchically porous structure, featuring large mesoporous channels, is particularly well suited for the accommodation of bulky drug molecules such as mitoxantrone, while preserving the crystallinity and morphology of the framework after loading. Direct comparison of biological performance with other reported systems should be made with caution, since cell models, assay conditions, and formulation parameters differ substantially across studies.

### 2.3. PEGylation and Stabilization Under Physiological Conditions

To improve the stability of the MTX@NU-1000 system under physiologically relevant conditions, the particles were coated with NH_2_-PEG-NH_2_, as described in the Section 3. PEG with a molecular weight of 2000 Da was selected to provide effective steric stabilization of the particles while minimizing potential pore blocking. This step was considered necessary because NU-1000 is known to be unstable in phosphate-containing media such as PBS 1× [31,32], which is widely used to mimic physiological conditions in biological assays. In this environment, the framework undergoes structural degradation, which may compromise the integrity of the carrier before its biological function can be exerted.

The protective effect of PEG was first evaluated on unloaded NU-1000 particles (Figure S4). As shown by PXRD and SEM, pristine NU-1000 loses its crystallinity and morphology after incubation in PBS 1× (Figure S4a,d), confirming the collapse of the framework under these conditions. PEG-coated NU-1000 retains the crystalline structure and morphology of the pristine material (Figure S4b,e), indicating that the coating process itself does not alter the MOF. Importantly, after exposure to PBS 1×, PEG@NU-1000 preserves both its crystallinity and particle morphology (Figure S4c,f), in clear contrast to the behavior of pristine NU-1000. This improved stability in phosphate-containing media constitutes an additional advantage of the NU-1000-based system over other Zr-based carriers whose evaluation under biologically relevant conditions may be limited by structural degradation.

**Figure 4:**
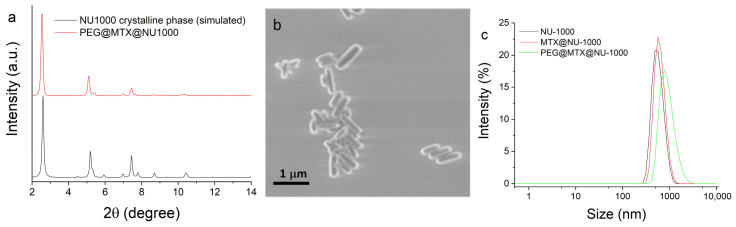
(**a**) PXRD pattern of PEG@MTX@NU-1000 compared with that of pristine NU-1000. (**b**) SEM micrograph of PEG@MTX@NU-1000 crystals. (**c**) DLS size distribution of PEG@MTX@NU-1000 particles dispersed in water, compared with pristine NU-1000 and MTX@NU-1000.

After confirming the protective role of PEG, the MTX-loaded particles were PEGylated and subsequently characterized (Figure 4). The PXRD pattern of PEG@MTX@NU-1000 (Figure 4a) shows that the crystalline structure of the MOF is preserved after coating, although with a slightly lower diffraction intensity than that of the uncoated material. Likewise, SEM images (Figure 4b) reveal that the particles retain their rod-like morphology and overall size after PEGylation. A slightly enhanced contrast at the particle edges is observed, which may be associated with changes in surface properties induced by the PEG coating. Some local particle clustering can also be observed in the SEM micrograph. This more compact appearance is attributed mainly to drying effects during SEM sample preparation and may be further favored by the presence of the PEG coating.

DLS measurements further support the presence of a PEG layer around the particles and indicate changes in their behavior in aqueous suspension. The average hydrodynamic diameter increases from 708 nm for MTX@NU-1000 to 952 nm after PEGylation, together with a broader size distribution (PDI ≈ 0.25), which is consistent with the formation of a hydrated polymer shell and/or increased interparticle interactions in solution. This interpretation is further supported by Zeta-potential measurements in water. Pristine NU-1000 and MTX@NU-1000 exhibit positive Zeta-potential values of approximately +18 and +16 mV, respectively, consistent with the positive Zeta-potential values reported for related zirconium-based MOFs in aqueous media [40,41]. After PEGylation, the Zeta-potential decreases markedly to about +3 mV, indicating effective surface shielding by PEG.

Although the exact mode of PEG attachment was not investigated in detail and direct spectroscopic confirmation of PEGylation (e.g., by FTIR or XPS) was not performed, the coating is most likely established through non-covalent interactions between NH_2_-PEG-NH_2_ and the external surface of NU-1000, including coordination of terminal amino groups to accessible Zr sites and hydrogen-bonding interactions with surface hydroxyl groups. In this context, PEG acts as a steric stabilizing shell rather than as a covalently bound modification, which is consistent with the preservation of the MOF crystalline structure together with the changes observed in hydrodynamic size and Zeta-potential, as well as the improved structural stability after coating.

### 2.4. Cytotoxicity of PEG@NU-1000 and Free Mitoxantrone

**Figure 5:**
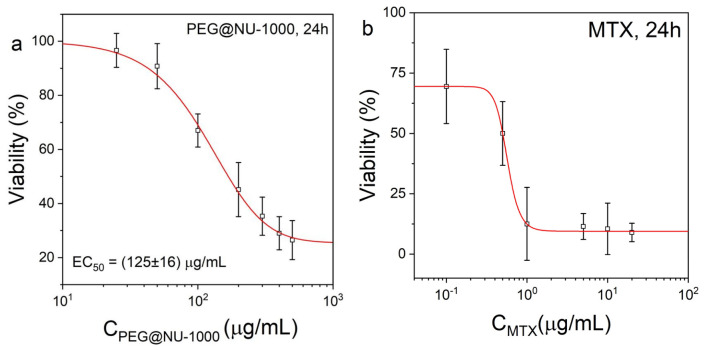
Cytotoxicity of (**a**) PEG@NU-1000 and (**b**) free mitoxantrone (MTX) in HeLa cells after 24 h of incubation, determined by the MTT assay. Data are presented as cell viability (%) as a function of concentration. The solid lines correspond to sigmoidal fits of the experimental data used to estimate EC_50_ values.

The cytotoxicity of PEG@NU-1000 was first evaluated in HeLa cells after 24 h of exposure (Figure 5a). The results show a typical dose–response behavior, with cell viability remaining close to 100% at low concentrations and gradually decreasing at higher doses. The data follow a sigmoidal trend, characteristic of biological response systems, from which an EC_50_ value of 125 ± 16 µg/mL was determined. These results indicate that PEG@NU-1000 exhibits low toxicity within the concentration range used in this work, confirming its suitability as a drug carrier. The decrease in viability observed at higher concentrations is consistent with nonspecific stress effects commonly reported for nanoparticulate systems at elevated doses [14,42].

The cytotoxic profile of free mitoxantrone was evaluated under the same conditions (Figure 5b). In contrast to PEG@NU-1000, MTX exhibits a much steeper dose–response curve, with a pronounced decrease in cell viability occurring within a narrow concentration range. An EC_50_ value of approximately 0.5–0.7 µg/mL can be estimated, indicating a markedly higher potency compared to the carrier material. At concentrations above 1 µg/mL, cell viability drops to values below 15%, reflecting the strong cytotoxic effect of MTX and defining the relevant concentration range for comparison with the encapsulated system.

### 2.5. Antiproliferative Activity of PEG@MTX@NU-1000

**Figure 6:**
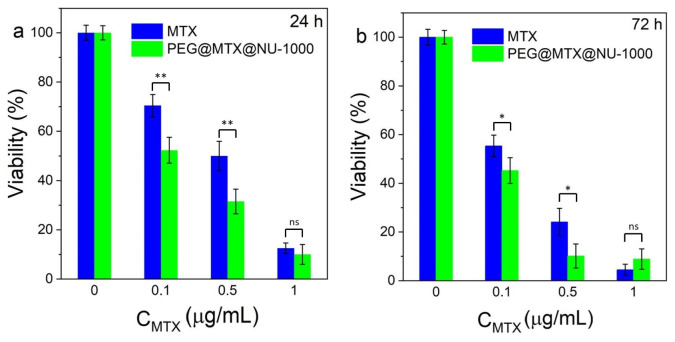
Cytotoxicity of free MTX and PEG@MTX@NU-1000 in HeLa cells after (**a**) 24 h and (**b**) 72 h of incubation, determined by the MTT assay. Cell viability (%) is plotted as a function of MTX concentration. For PEG@MTX@NU-1000, concentrations are expressed as equivalent MTX content based on the loading capacity. Statistical significance between free MTX and PEG@MTX@NU-1000 at each concentration was evaluated using a two-tailed Welch’s *t*-test (* *p* < 0.05; ** *p* < 0.01; ns, not significant).

The cytotoxic effect of PEG@MTX@NU-1000 was evaluated and compared with that of free MTX at equivalent drug concentrations (Figure 6). At 24 h, the encapsulated system exhibits a significantly enhanced antiproliferative effect compared to the free drug. For instance, at 0.5 µg/mL, cell viabilities of 50.2 ± 5.9% and 30.8 ± 4.8% were obtained for free MTX and PEG@MTX@NU-1000, respectively. Importantly, the corresponding concentration of MOF composite (~1.16 µg/mL, calculated from a loading capacity of 43 wt%) is far below the EC_50_ determined for PEG@NU-1000 (125 µg/mL), indicating that the observed cytotoxicity arises predominantly from the drug and not from the carrier. Statistical analysis confirmed that the differences between free MTX and PEG@MTX@NU-1000 were significant at 0.1 and 0.5 µg/mL after 24 h of incubation, whereas no significant difference was observed at 1.0 µg/mL.

The enhanced effect observed at early incubation times suggests that encapsulation modifies the effective interaction of MTX with the cellular environment, leading to a more pronounced antiproliferative response. Possible non-exclusive contributors to this behavior include an increase in local MTX availability at the cell interface, a partial contribution from particle-associated uptake or close particle-cell contact, and a potential protective effect of encapsulation on MTX availability prior to cell interaction. However, the relative contribution of drug release, particle uptake, particle-cell contact, and local drug availability remains to be clarified. After 72 h of incubation, statistically significant differences between free MTX and PEG@MTX@NU-1000 were maintained at 0.1 and 0.5 µg/mL, whereas the difference at 1.0 µg/mL was not significant. Nevertheless, the overall reduction in the gap between both treatments at longer incubation times indicates that free MTX is also able to reach its full cytotoxic effect upon prolonged exposure.

Overall, these results demonstrate that PEG@MTX@NU-1000 enhances the short-term cytotoxic activity of MTX, highlighting the potential of this system as an efficient drug delivery platform. However, since the biological evaluation was performed only in HeLa cells, potential variability across different cell types cannot be excluded and broader validation in additional models will be necessary to assess the generality of this behavior.

## 3. Materials and Methods

### 3.1. Materials and Characterization Methods

NH_2_-Polyethylene glycol-NH_2_ (PEG, 2000 Da) was purchased from RAPP Polymere (Tuebingen, Germany). 4,4′,4″,4‴-(pyrene-1,3,6,8-tetrayl) tetrabenzoic acid (H_4_-TBAPy) was purchased from BLDPharm (Shanghai, China). Zirconyl chloride octahydrate (ZrOCl_2_·8H_2_O) and benzoic acid were purchased from Sigma-Aldrich (Darmstadt, Germany). Mitoxantrone was purchased as the dihydrochloride from Enzo Life Sciences (Farmingdale, NY, USA). Other reagents and solvents were purchased as analytical grade and used without further purification.

Powder X-ray diffraction (PXRD) analysis was performed on approximately 10–15 mg of each sample at room temperature using a Bruker D8 Discover diffractometer at 50 kV and 1000 mA with Cu kα radiation (λ = 1.5418 Ǻ), in the range 2°–14° (2θ) in still mode, with a step time of 300 s. The distance between the sample and the detector was 30 cm.

The shape and size of the MOF particles were examined by scanning electron microscopy (SEM), using a ZEISS Gemini 300 instrument (Oberkochen, Germany). Ultraviolet-visible (UV-Vis) absorption spectra were recorded for drug and drug-MOF samples using an Agilent Technologies Cary Series spectrophotometer (Santa Clara, CA, USA) in the 400–800 nm range. Dynamic Light Scattering (DLS) and Zeta-potential measurements were performed using a Malvern Panalytical Zetasizer Ultra-Red instrument (Malvern, UK) at 25 °C. For DLS measurements, the samples were dispersed in water at a MOF concentration of 0.01 mg/mL and analyzed using 12 scans per measurement.

Thermogravimetry (TGA) analysis was performed on approximately 1–2 mg of each sample over a temperature range of 0–1000 °C using a TA Instruments TGA Discovery instrument (New Castle, DE, USA) under a linear setting and a temperature ramp of 1 °C min^−1^.

### 3.2. NU-1000 Synthesis

NU-1000 was synthesized via a solvothermal reaction of zirconium chloride with H_4_-TBAPy ligands, following the literature protocol [43] with some variations. Briefly, 98 mg (0.3 mmol) of ZrOCl_2_·8H_2_O and 2 g (16.38 mmol) of benzoic acid were dissolved in 8 mL of dimethylformamide (DMF) and heated at 100 °C in an oil bath for 1 h under magnetic stirring at 270 rpm. Meanwhile, 40 mg (0.06 mmol) of H_4_-TBAPy were dissolved in 4 mL of DMF, to which 40 μL (0.52 mmol) of trifluoroacetic acid (TFA) were added, and the solution was sonicated for 10 min. Then, the ligand solution was quickly poured into the metal solution under magnetic stirring at 270 rpm and maintained at 100 °C for an additional hour.

The NU-1000 particles were then washed three times with DMF, with 30 min sonication between washes, and underwent an acid treatment with 0.5 mL of 8 M HCl overnight. After that, they were washed twice with DMF and three times with MeOH, with 30 min sonication between MeOH washes, and finally resuspended in 5 mL of MeOH.

### 3.3. MTX@NU-1000 Inclusion Assays

An amount of 5 mg of MTX was dissolved in 10 mL of water. The absorbance spectrum of this solution was recorded prior to the addition of NU-1000. Subsequently, 10 mg of NU-1000 were added and the mixture was gently stirred for 24 h. At the end of the experiment, the suspension was centrifuged at 2.8 × 10^3^ g (6500 rpm, rotor radius = 6 cm) for 15 min, and the absorbance spectrum of the supernatant was measured by UV-Vis spectroscopy to quantify unencapsulated MTX. The assay was then repeated under identical conditions to follow the inclusion kinetics. In this case, stirring was periodically stopped during the first three hours, and samples were taken at different times. Each sample was centrifuged under the same conditions (2.8 × 10^3^ g, 15 min), and the absorbance spectrum of the supernatant was recorded. Finally, once the inclusion assays were completed, the MTX@NU-1000 particles were collected by centrifugation and washed with water several times until the supernatant became colorless.

### 3.4. PEG@MTX@NU-1000 Particles Preparation

10 mg of MTX@NU-1000 particles and 5 mg of NH_2_-PEG-NH_2_ were mixed in water and gently stirred overnight. The resulting product was then collected by centrifugation at 2.8 × 10^3^ g (6500 rpm, rotor radius = 6 cm) for 15 min and washed three times with anhydrous ethanol.

### 3.5. Cytotoxicity Assays

Cytotoxicity assays were performed using HeLa cells seeded in 96-well plates. Briefly, 5000 cells per well were plated in 200 µL of complete growth medium, DMEM (Gibco) 4.5 g/L glucose with 10% fetal bovine serum (FBS), and incubated at 37 °C under a 5% CO_2_ atmosphere for 24 h to allow cell attachment. After this period, the culture medium was removed and replaced with fresh phenol red-free and fetal bovine serum-free medium containing the corresponding treatments: PEG@NU-1000, free mitoxantrone (MTX), or PEG@MTX@NU-1000. For the composite, the MTX concentration was calculated based on the loading capacity (LC) determined experimentally. Each plate included four blank wells containing medium only. The remaining wells were treated with the following concentrations (four replicates per condition):

1. PEG@NU-1000: 0, 25, 50, 100, 200, 300, 400, 500, and 600 µg/mL.
2. MTX: 0, 0.1, 0.5, 1, 5, 10, and 20 µg/mL.
3. PEG@MTX@NU-1000: concentrations equivalent to 0, 0.1, 0.5, 1, 5, 10, and 20 µg/mL of MTX.

Cells were exposed to the treatments for 24 and 72 h under standard incubation conditions (37 °C, 5% CO_2_). After each incubation period, 20 µL of MTT solution (0.5 mg/mL in culture medium) were added to each well, and the plates were incubated for an additional 4 h. Subsequently, the supernatant was carefully removed, and 150 µL of dimethyl sulfoxide (DMSO) were added to dissolve the formazan crystals. Absorbance was measured at 490 nm using a microplate reader (Cytation 1). The cytotoxicity data shown in Figure 5 and Figure 6 were obtained from four independent experiments. Statistical comparison between free MTX and PEG@MTX@NU-1000 at each concentration and incubation time was performed using a two-tailed Welch’s *t*-test. Differences were considered statistically significant at *p* < 0.05.

## 4. Conclusions

This work demonstrates that the zirconium-based MOF NU-1000 is a suitable platform for the delivery of the antineoplastic drug mitoxantrone. The material exhibits a high loading capacity (~40–43 wt%) while preserving the crystallinity and morphology of the framework after drug incorporation. The combined PXRD, SEM, DLS, UV-Vis, and TGA results support the successful formation of the MTX@NU-1000 composite and indicate that the drug is predominantly accommodated within the internal pore system of the MOF. PEGylation was shown to be an effective strategy to improve the stability of the system under physiologically relevant conditions. In contrast to pristine NU-1000, which undergoes structural degradation in PBS, PEG-coated particles retain their crystallinity and morphology after exposure to this medium. The increase in hydrodynamic diameter and the marked decrease in Zeta-potential after PEGylation further support the formation of a stabilizing PEG layer around the MOF particles. Finally, cytotoxicity assays in HeLa cells revealed that PEG@NU-1000 is only weakly toxic within the concentration range used in this work, whereas PEG@MTX@NU-1000 exhibits a more pronounced antiproliferative effect than free MTX at short incubation times. Taken together, these results highlight the potential of PEGylated NU-1000 as a promising carrier for mitoxantrone delivery.

## Data Availability

The original contributions presented in this study are included in the article and Supplementary Materials. Further inquiries can be directed to the corresponding authors.

## Supplementary Materials

The following supporting information can be downloaded at https://www.mdpi.com/article/10.3390/ijms27114857/s1.

## Abbreviations

The following abbreviations are used in this manuscript:

MOFs	Metal–organic frameworks
MTX	Mitoxantrone
PEG	Polyethylene glycol
PXRD	Powder X-ray diffraction
SEM	Scanning electron microscopy
DLS	Dynamic light scattering
UV-Vis	Ultraviolet-visible spectroscopy
TGA	Thermogravimetric analysis
PBS	Phosphate-buffered saline
PDI	Polydispersity index
LC	Loading capacity
