# Supplementary material for: Use of the MOF NU-1000 as a Drug Delivery System for the Antineoplastic Drug Mitoxantrone
Source: Int J Mol Sci. 2026 May 28;27(11):4857. doi: 10.3390/ijms27114857 (PMC13256363; doi:10.3390/ijms27114857)
Supplement: Supplementary file 1 [file ijms-27-04857-s001.zip › ijms-4288870-supplementary.pdf]

Supplementary Material

Use of the MOF NU-1000 as a Drug Delivery System for the Antineoplastic Drug Mitoxantrone

Daniel R. Alfonso[1], Francisco G. Moscoso[1], David Rodríguez-Lucena[1,2*], Javier Roales[1], Carolina Carrillo-Carrión[3], María Victoria Cascajo-Almenara[4], Carlos Santos-Ocaña[4] and José M. Pedrosa[1*]

[1] *Center for Nanoscience and Sustainable Technologies (CNATS), Department of Physical, Chemical and Natural Systems, Universidad Pablo de Olavide, 41013 Sevilla, Spain.*

[2] *Departamento de Química Orgánica y Farmacéutica, Facultad de Farmacia, Universidad de Sevilla, 41012 Sevilla, Spain*

[3] *Institute for Chemical Research (IIQ), CSIC-University of Seville, 41092 Sevilla, Spain*

[4] *Andalusian Center for Developmental Biology, University Pablo de Olavide, CIBERER, 41013 Sevilla, Spain.*

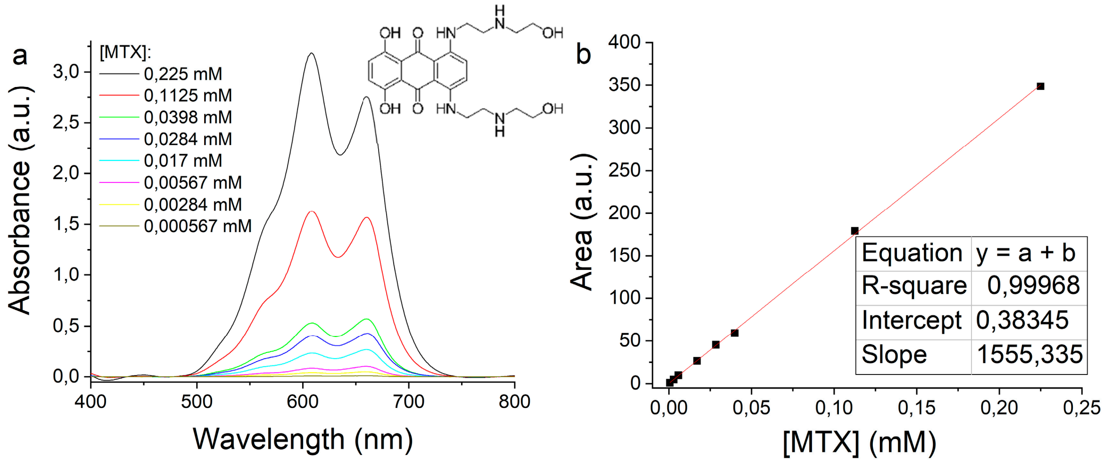

Figure S1. (a) UV-Vis absorption spectra of mitoxantrone (MTX; molecular structure shown in the inset) in phosphate-buffered saline (PBS 1×, pH 7.4) at different concentrations. (b) Corresponding calibration curve obtained from the integrated area of the absorption bands in the 500–750 nm range.

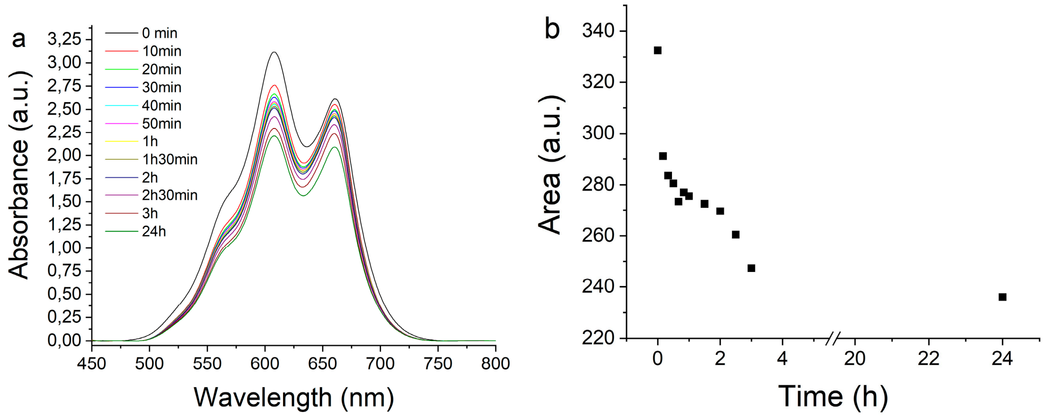

Figure S2. (a) UV–Vis absorption spectra of the supernatant at different times during MTX encapsulation in NU-1000. (b) Time evolution of the integrated area of the absorption bands calculated from the spectra shown in (a).

Table S1. Percentage of MTX uptake from solution at representative time points during the loading of NU-1000.

Time	MTX Uptake (%)
10 min	40.4
30 min	49.0
1 h	53.1
3 h	69.4
24 h	86.0

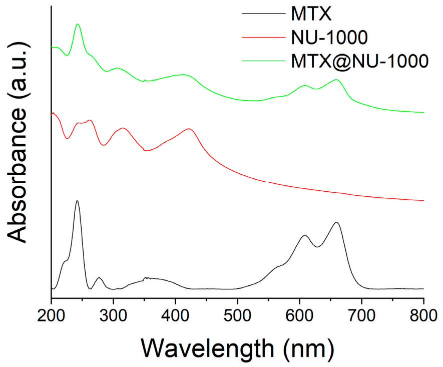

Figure S3. UV–Vis absorption spectra of MTX in aqueous solution, NU-1000 in aqueous suspension, and MTX@NU-1000 in aqueous suspension, shown together for comparison.

TGA calculation. The loading capacity was also determined from TGA analysis. First, the molar ratio between zirconium and the organic linker (n*Zr*/n*TBAPy*) was calculated from the TGA curve of pristine NU-1000 (Figure 3). This calculation assumes complete decomposition of the organic fraction, with the remaining mass corresponding exclusively to the inorganic residue in the form of ZrO_2.

The molar ratio was calculated using the following expression:

$$\frac{n_{Zr}}{n_{TBAPy}} = \frac{\%ZrO_2}{\%TBAPy}\frac{Mw(TBAPy)}{Mw(ZrO_2)} \qquad \text{(S1)}$$

where $\%ZrO_2$ corresponds to the residual mass at high temperature (>500 °C), and %*TBAPy* corresponds to the mass loss attributed to the decomposition of the organic linker. $Mw(ZrO_2)$ and *Mw*(*TBAPy*) are the molar weights of ZrO_2 and the TBAPy linker, respectively.

From this analysis, a molar ratio n*Zr*/n*TBAPy* = 3.7 was obtained. The incorporation of MTX was evaluated by comparing the TGA curve of MTX@NU-1000 with that of pristine NU-1000. Assuming that the Zr/TBAPy ratio remains constant after MTX loading, the additional mass loss observed in MTX@NU-1000 was attributed to the presence of MTX.

Under these assumptions, application of Equation (S1) to the TGA data of the composite yielded a value of %*TBAPy* = 36.7 wt%. The remaining organic mass was attributed to MTX, resulting in a MTX content of 28.3 wt%. Based on this value, the loading capacity was estimated to be 39 wt%. This value corresponds to the loading capacity expressed as mg of MTX per mg of NU-1000.

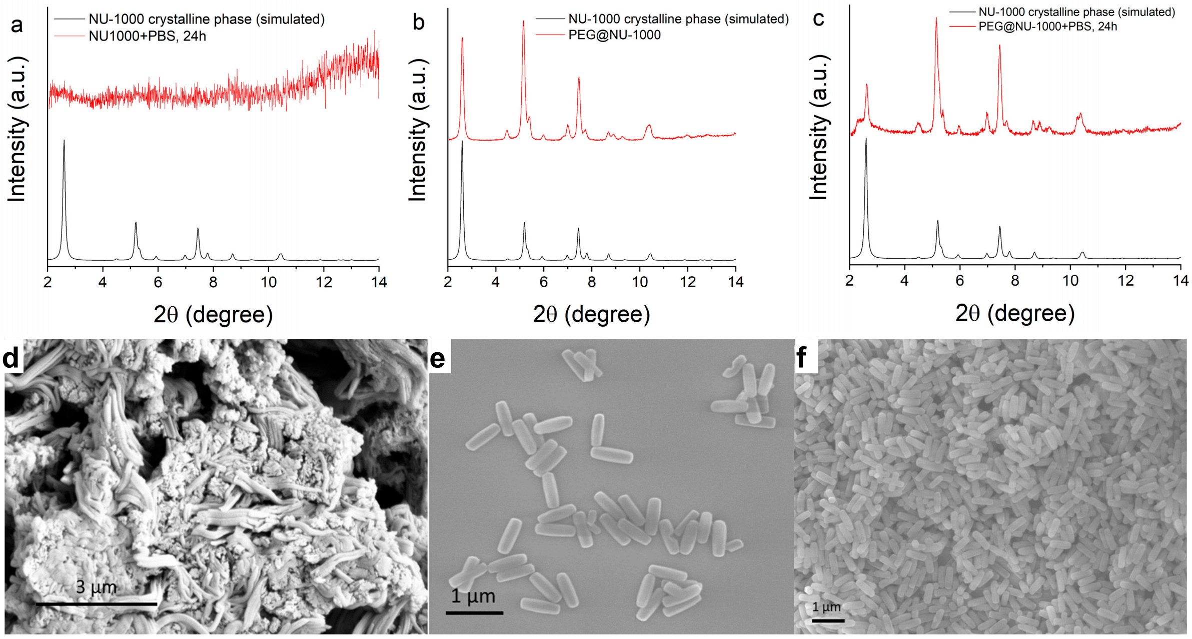

Figure S4. (a) PXRD pattern of NU-1000 after incubation in PBS 1× for 24 h. (b) PXRD pattern of PEG@NU-1000. (c) PXRD pattern of PEG@NU-1000 after incubation in PBS 1× for 24 h. (d) SEM micrograph of NU-1000 after incubation in PBS 1× for 24 h. (e) SEM micrograph of PEG@NU-1000. (f) SEM micrograph of PEG@NU-1000 after incubation in PBS 1× for 24 h.
